# Lady Cowdray's Great Ideal

**Published:** 1921-01-22

**Authors:** 


### Lady Cowdray's Great Ideal.

In commenting last October on Lord and Lady Cowdray's munificent gift of a "Nursing Centre for the Empire" we ventured to forecast the erection on the vacant site behind the club premises in Cavendish Square of a building "worthy to be called the centre of the nursing profession." It seemed then but a very remote prospect, for architectural developments must needs give place to the exigencies of administration and the slow building up of a profession not very opulent even in its most prosperous undertakings.

The dream has, however, come true. We understand that Lord and Lady Cowdray have signified their intention of crowning their gifts to the nursing profession with a College Building suitable to the immense and ever-increasing developments of the College activities; that plans have been prepared, and that the work will be put in hand as soon as possible, though obviously some considerable time, perhaps years, must of necessity elapse before the complete scheme can be carried out.

The task of commemorating the nurses of the Empire is one into which Lady Cowdray has thrown her heart and soul. It is a great opportunity, which she has embraced with an enthusiasm, nobly seconded by her husband, and the effects of their joint munificence (may we not say their power of vision?) will remain a monument for many generations to come of far-sighted liberality. It will add welcome distinction to the part of London long appropriated to doctors' and nurses' interests.

Beauty is the last consideration, as a rule, in catering for the needs of the nursing profession. Yet in truth beauty, light, and colour are among the crying needs of a profession whose work so often leads its members into drab and ugly surroundings, depleting their vitality till their starved artistic instincts call out insistently to be satisfied. Space for the assembling of nurses from every part of Great Britain and from the overseas dominions will in itself bring with it a sense of corporate strength. Other parts of the great scheme will gradually unfold themselves as time goes on. Among the many possibilities, we look forward to the banners of varied groups of nurses, a great organ to lead massed choirs from the centres, and many inspiring functions destined to bring nurses in their separate vocations that sense of unity for which they crave and which they shall no longer lack.

It is a great ideal, destined perhaps to effect more even than the donors can at this moment realise.

